# 47982 Reversing the resistance to chemotherapy in White and Black patients with triple negative breast cancer (TNBC).

**DOI:** 10.1017/cts.2021.652

**Published:** 2021-03-31

**Authors:** Shipra Gandhi, Thaer Khoury, Kristopher Attwood, Song Yao, Christine Ambrosone, Kazuaki Takabe, Pawel Kalinski

**Affiliations:** Roswell Park Comprehensive Cancer Center

## Abstract

ABSTRACT IMPACT: Reversing tumor microenvironment (TME) immunosuppression will help to increase the overall efficacy of treatment of chemo-resistant triple negative breast cancer (TNBC) and mitigate racial disparities in treatment response. OBJECTIVES/GOALS: We have developed an ex-vivo whole tissue culture model to test the feasibility of reversing local immunosuppression in TME by chemokine modulatory (CKM) regimen. Our current objective is to analyze the molecular changes in CKM-treated chemoresistant TNBC from White and Black women and identify factors determining response to CKM. METHODS/STUDY POPULATION: Freshly resected residual TNBC from 20 White and 20 Black women ≥18 yrs old treated with neoadjuvant chemotherapy (NAC) will be procured. Tumor explants will be prepared & cultured in the absence and presence of CKM (Interferon-ð 〉1/4, TLR3 agonist rintatolimod and COX-2 inhibitor celecoxib). Chemokines implicated in cytotoxic T-lymphocyte (CTL)- & MDSC/ Treg attraction will be analyzed using Taqman & ELISA. We will have 80% power to detect a 0.7 standard deviation difference in chemokines between untreated & treated samples within and between cohorts using ANCOVA. Bulk RNA sequencing will be performed on both untreated & treated samples from CKM responding (highest aggregate increase in CTL- and highest decrease in Treg/MDSC-favoring chemokines in the top quartile) and non-responding (bottom quartile) tissues. RESULTS/ANTICIPATED RESULTS: Our preliminary data show that Black patients (pts) with breast cancer (BC) have an immunosuppressive TME associated with poor outcomes. This is similar to other existing literature showing that Black pts with BC have less favorable and more unfavorable chemokines in the TME. We anticipate the chemokine changes with CKM treatment will be larger in the Black cohort given their ability to elicit a robust inflammatory response. Therefore, we expect that CKM treatment will result in favorable TME in both groups and improve outcomes in TNBC, which has the worst prognosis of all subtypes, eliminating a key area of disparity in BC. The proposed transcriptome analysis will help identify key gene networks involved in response to CKM treatment and guide modulating the targets for non-responsiveness to improve efficacy of CKM. DISCUSSION/SIGNIFICANCE OF FINDINGS: Pts with residual disease (RD) after NAC have a 3-yr overall survival 68% vs. 94% for pts with complete response. Blacks have a higher incidence of TNBC with more likelihood of RD & mortality. We anticipate that the existing TME differences can be abrogated by our current CKM regimen or via developing an alternative CKM regimen optimized for Black pts.

